# Genome-Wide Meta-Analysis Identifies Regions on 7p21 (*AHR*) and 15q24 (*CYP1A2*) As Determinants of Habitual Caffeine Consumption

**DOI:** 10.1371/journal.pgen.1002033

**Published:** 2011-04-07

**Authors:** Marilyn C. Cornelis, Keri L. Monda, Kai Yu, Nina Paynter, Elizabeth M. Azzato, Siiri N. Bennett, Sonja I. Berndt, Eric Boerwinkle, Stephen Chanock, Nilanjan Chatterjee, David Couper, Gary Curhan, Gerardo Heiss, Frank B. Hu, David J. Hunter, Kevin Jacobs, Majken K. Jensen, Peter Kraft, Maria Teresa Landi, Jennifer A. Nettleton, Mark P. Purdue, Preetha Rajaraman, Eric B. Rimm, Lynda M. Rose, Nathaniel Rothman, Debra Silverman, Rachael Stolzenberg-Solomon, Amy Subar, Meredith Yeager, Daniel I. Chasman, Rob M. van Dam, Neil E. Caporaso

**Affiliations:** 1 Department of Nutrition, Harvard School of Public Health, Boston, Massachusetts, United States of America; 2 Department of Epidemiology, The University of North Carolina at Chapel Hill, Chapel Hill, North Carolina, United States of America; 3 Division of Cancer Epidemiology and Genetics, National Cancer Institute, National Institutes of Health, Bethesda, Maryland, United States of America; 4 Brigham and Women's Hospital, Boston, Massachusetts, United States of America; 5 Collaborative Health Studies Coordinating Center, University of Washington, Seattle, Washington, United States of America; 6 Division of Epidemiology, Human Genetics, and Environmental Sciences, The University of Texas Health Science Center at Houston, Houston, Texas, United States of America; 7 Department of Biostatistics, Collaborative Studies Coordinating Center, The University of North Carolina at Chapel Hill, Chapel Hill, North Carolina, United States of America; 8 Brigham and Women's Hospital, Harvard Medical School, Boston, Massachusetts, United States of America; 9 Program in Molecular and Genetic Epidemiology, Harvard School of Public Health, Boston, Massachusetts, United States of America; 10 Department of Epidemiology and Public Health and Department of Medicine, Yong Loo Lin School of Medicine, National University of Singapore, Singapore, Singapore; Georgia Institute of Technology, United States of America

## Abstract

We report the first genome-wide association study of habitual caffeine intake. We included 47,341 individuals of European descent based on five population-based studies within the United States. In a meta-analysis adjusted for age, sex, smoking, and eigenvectors of population variation, two loci achieved genome-wide significance: 7p21 (*P* = 2.4×10^−19^), near *AHR*, and 15q24 (*P* = 5.2×10^−14^), between *CYP1A1* and *CYP1A2*. Both the *AHR* and *CYP1A2* genes are biologically plausible candidates as *CYP1A2* metabolizes caffeine and *AHR* regulates *CYP1A2*.

## Author Summary

Caffeine is the most widely consumed psychoactive substance in the world. Although demographic and social factors have been linked to habitual caffeine consumption, twin studies report a large heritable component. Through a comprehensive search of the human genome involving over 40,000 participants, we discovered two loci associated with habitual caffeine consumption: the first near *AHR* and the second between *CYP1A1* and *CYP1A2*. Both the *AHR* and *CYP1A2* genes are biologically plausible candidates, as *CYP1A2* metabolizes caffeine and *AHR* regulates *CYP1A2*. Caffeine intake has been associated with manifold physiologic effects and both detrimental and beneficial health outcomes. Knowledge of the genetic determinants of caffeine intake may provide insight into underlying mechanisms and may provide ways to study the potential health effects of caffeine more comprehensively.

## Introduction

Caffeine (1,3,7-trimethylxanthine) is the most widely consumed psychoactive substance in the world with nearly 90% of adults reporting regular consumption of caffeine-containing beverages and foods [1], [2]. Although demographic and social factors have been linked to habitual caffeine consumption, twin studies report heritability estimates between 43 and 58% for caffeine use; 77% for heavy use, and 45, 40, and 35%, respectively, for caffeine toxicity, tolerance and withdrawal symptoms [3]. Genetic association studies focused on candidate genes related to the pharmacokinetic and pharmacodynamic properties of caffeine have identified genes encoding cytochrome P-450 (CYP)1A2, as the primary enzyme involved in caffeine metabolism [3], [4]. The genome-wide association approach has emerged as a powerful means for discovering novel loci related to habitual use of a second stimulant, tobacco [5], but has not yet clearly identified genes for other common behavioral traits, including caffeine consumption. To comprehensively examine the influence of common genetic variation on habitual caffeine consumption behavior we undertook a meta-analysis of genome-wide association studies (GWAS) from population-based cohorts. Our study confirms the important roles of *CYP1A2* and *AHR* in determining caffeine intake, thus supporting the utility of the GWAS approach to the discovery of loci linked to this complex behavioral trait.

## Results

We performed a meta-analysis of 47,341 individuals of European descent, derived from five studies within the US, the Atherosclerosis Risk in Communities (ARIC, N = 8,945) Study, the Prostate, Lung, Colorectal, and Ovarian Cancer Screening Trial (PLCO, N = 4,942), the Nurses' Health Study (NHS, N = 6,774), the Health Professionals Follow-Up Study (HPFS, N = 4,023), and the Women's Genome Health Study (WGHS, N = 22,658). Sample characteristics are presented in Table 1. Caffeine intake was assessed using semi-quantitative food frequency questionnaires (FFQ) that included questions on the consumption of caffeinated coffee, tea, soft drinks, and chocolate.

**Table 1:** Descriptive characteristics of studies participating in meta-analysis.*

Study	Description	N	Female, %	Age, years	Caffeine, mg/day	Current smokers, %	Platform
ARIC	Cohort	8,945	52.8	54.3 (5.7)	332.9 (311.1)	24.4	Affymetrix 6.0
PLCO	Cohort: nested case-control**	4,942	23.5	67.7 (5.4)	491.1 (494.1)	22.1	Illumina 240KIllumina 310KIllumina 550kIllumina 610Q
NHS T2D	Cohort: nested T2D case-control	3,135	100	51.1 (10.5)	284.5 (206.3)	14.8	Affymetrix 6.0
NHS CHD	Cohort: nested CHD case-control	1,102	100	53.5(10.6)	316.7 (218.0)	30.0	Affymetrix 6.0
NHS KS	Cohort: nested KS case-control	488	100	47.7 (11.7)	264.4 (203.6)	15.3	Illumina 610Q
NHS BrC	Cohort: nested BrC case-control	2,049	100	52.3 (9.6)	286.5 (204.0)	15.6	Illumina 550k
HPFS T2D	Cohort: nested T2D case-control	2,381	0	55.5 (8.4)	250.9 (227.6)	7.6	Affymetrix 6.0
HPFS CHD	Cohort: nested CHD case-control	1,099	0	56.7 (8.7)	243.2 (230.7)	9.9	Affymetrix 6.0
HPFS KS	Cohort: nested KS case-control	543	0	48.8 (6.8)	230.5 (241.6)	6.4	Illumina 610Q
WGHS	Cohort	22,658	100	54.7 (7.1)	298.5 (232.9)	11.5	Illumina HumanHap300 Duo+
**Total**		47,341					

*Values are mean (standard deviation) for age and caffeine; percent for female and current smokers.

**Includes samples from prostate cancer case-control (n = 1885), bladder cancer case-control (n = 572), glioma case-control (n = 3), lung cancer case-control (n = 1758), pancreatic cancer case-control (n = 299), renal cancer case-control study (n = 271).

**Figure 1:**
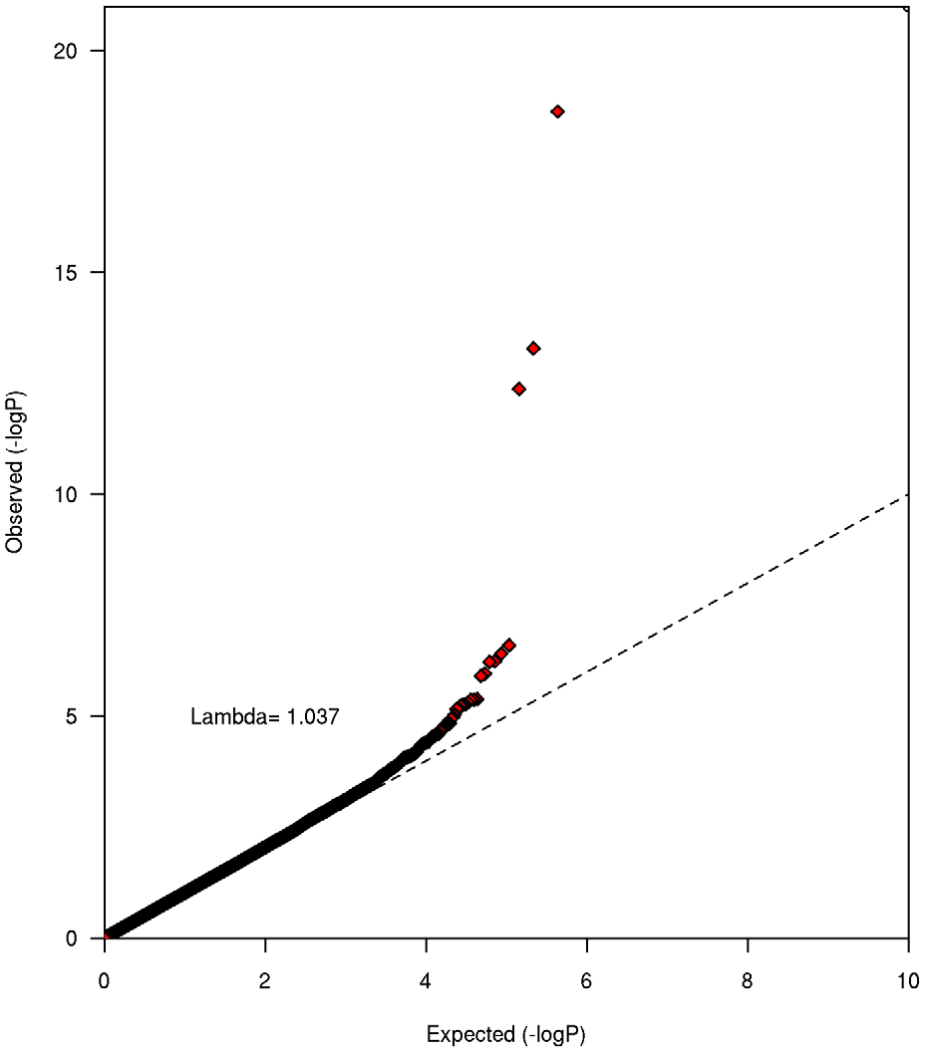
QQ plot for the genome-wide meta-analysis of caffeine consumption.

Study-level genomic inflation factors (λ) were low ranging from 1.00 (PLCO) to 1.03 (HPFS), suggesting that population stratification was well controlled (Figure S1). A total of 433,781 imputed and genotyped SNPs passed our stringent criteria for the meta-analysis. Test statistic inflation at the meta-analysis level revealed no evidence of notable underlying population substructure (λ = 1.04, Figure 1).

**Figure 2:**
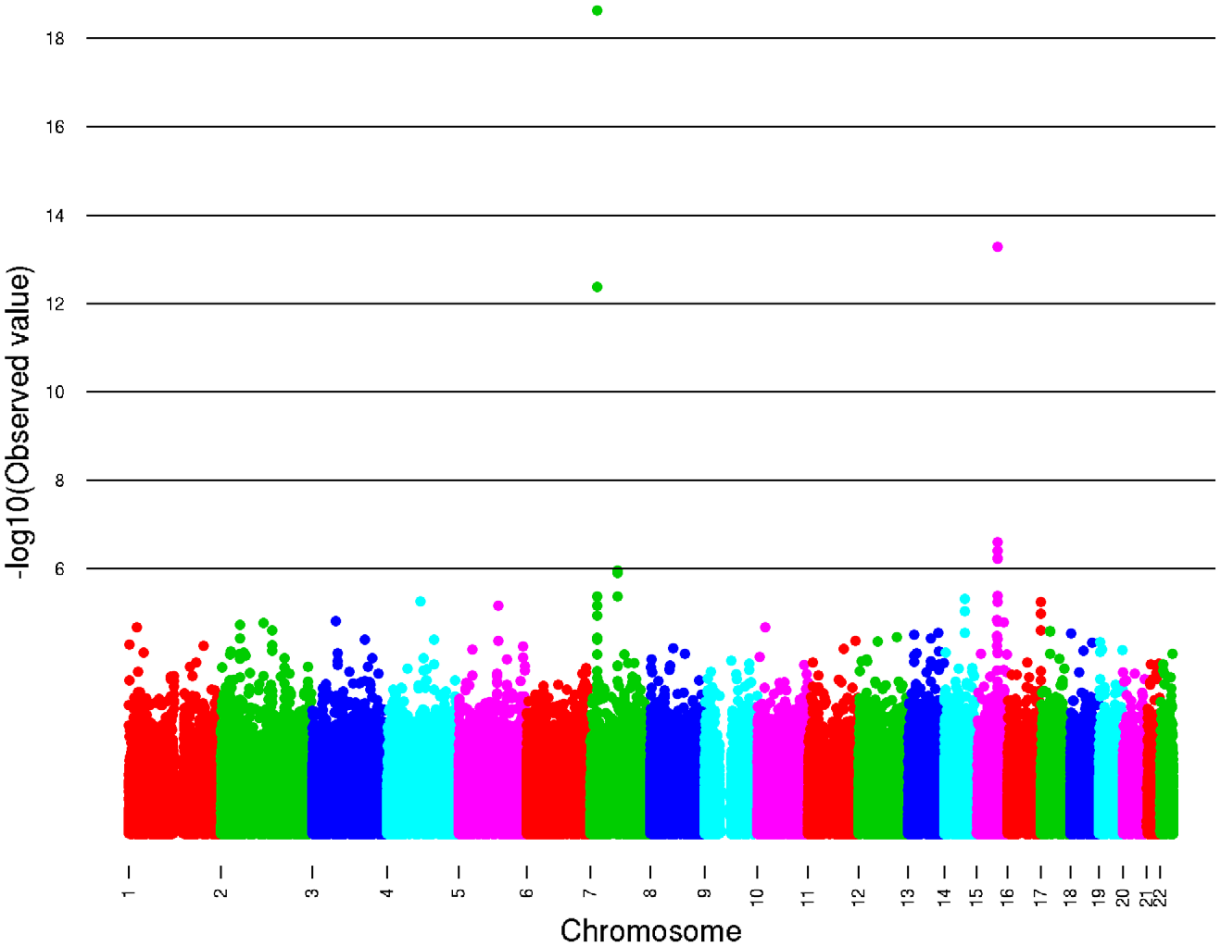
The –log10 P-plots for the genome-wide meta-analysis of caffeine consumption.

**Figure 3:**
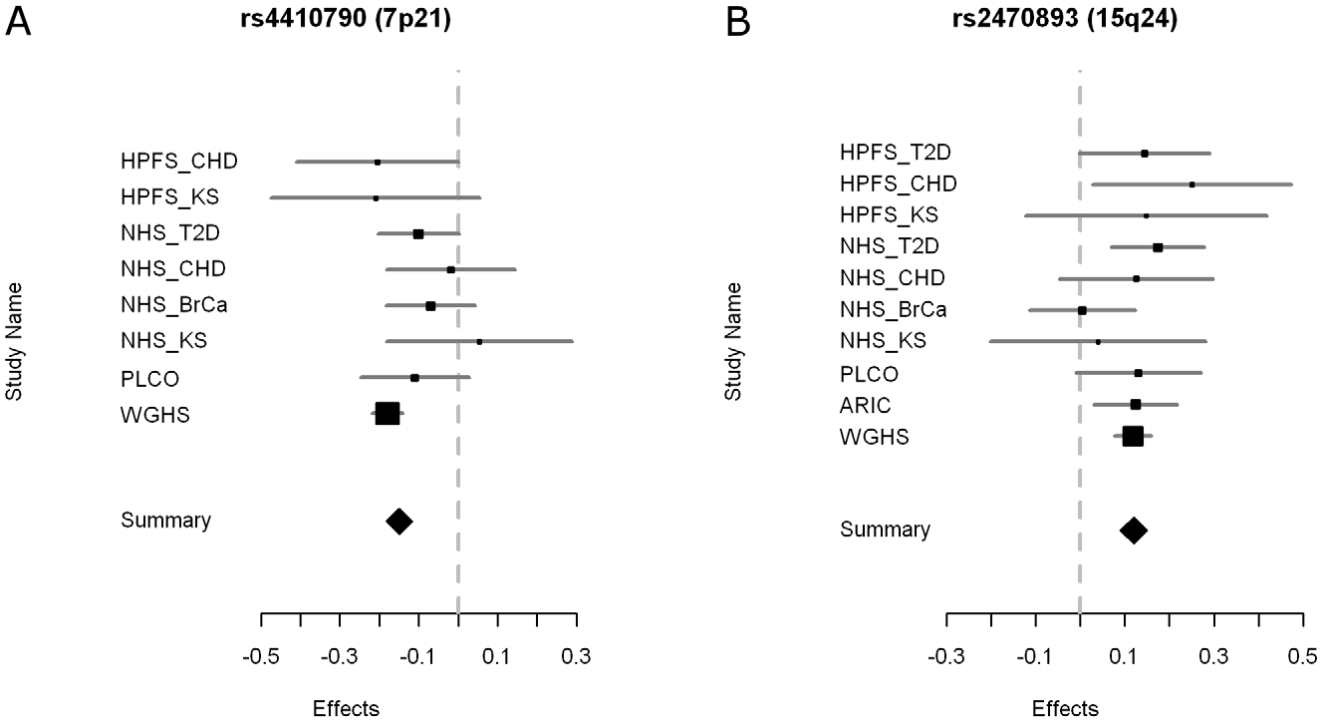
Forest plots of the meta-analysis for the two caffeine-associated loci. A) rs4410790 and B) rs2470893. The contributing effect from each study is shown by a square, with confidence intervals indicated by horizontal lines. The contributing weight of each study to the meta-analysis is indicated by the size of the square. The meta-analysis estimate is shown at the bottom of each graph.

Two loci reached genome-wide significance with no evidence for significant between- study heterogeneity (Table 2, Figure 2 and Figure 3, Table S1). The strongest associated SNP (rs4410790, *P* = 2.4×10^−19^, Figure S2) is located at 7p21, 54 kb upstream of *AHR* (aryl hydrocarbon receptor). The second strongest associated SNP (rs2470893, *P* = 5.2×10^−14^, Figure S2) mapped to 15q24 within the bidirectional promoter of the *CYP1A1-CYP1A2* locus [6], [7]. A synonymous coding SNP (rs2472304, *P* = 2.5×10^−7^) in *CYP1A2* exon 7 that was highly correlated with 6 other SNPs but not correlated with rs2470893 (r^2^ = 0.18, HapMap CEU) was amongst the highest ranked loci in our meta-analysis (Table 2). Although we only considered variants that were imputed with high probability, we also conducted a sensitivity analysis restricting our sampling to individuals with genotyped data (Table 2). Regression coefficients remained essentially unchanged, but *P*-values were less significant reflecting the reduced sample size (rs4410790: *P* = 4.0×10^−18^; rs2470893 *P* = 9.5×10^−8^). Similar results were also observed when men and women were examined separately (Table S2). Had the analysis been performed instead by discovery at genome-wide significance (*P*<5×10^−8^) in the WGHS followed by replication in meta-analysis of the remaining cohorts, only SNPs at the same loci would have met Bonferroni corrected standards of significance. In a *post-hoc* investigation of study heterogeneity in which we compared WGHS to the remaining studies combined, there was significant heterogeneity for rs4410790 (*P* = 0.01), although this could be attributable to chance.

**Table 2:** Genome-wide meta-analytic results for caffeine consumption (*P*<10^−6^).

Index SNP	Chr	Position (NCBI 36)	Closest gene(s) (±100 kb)	Total SNPs* (*P*<1×10^−3^)	EA	EAF	Imputed and Genotyped				Genotyped		
							N	β (SE)	*P*	*P*_het_ **	N	β (SE)	*P*
rs4410790	7	17251102	*AHR*	1	T	0.38	36013	−0.15 (0.02)	2.4×10^−19^	0.14	25738	−0.16 (0.02)	4.0×10^−18^
rs2470893	15	72806502	*LMAN1L, EDC3, CYP1A2, CYP1A1, CSK*	1	T	0.31	47341	0.12 (0.02)	5.2×10^−14^	0.68	25738	0.10 (0.02)	9.5×10^−8^
rs2472304	15	72831291	*CYP1A2*	4	A	0.65	47325	0.08 (0.01)	2.5×10^−7^	0.06	30663	0.07 (0.02)	3.2×10^−4^
rs6495122	15	72912698	*ULK3, SCAMP2, MP1, LMAN1L, CYP1A2, CSK, COX5A, CPLX3, C14orf17*	1	A	0.43	47341	−0.07 (0.01)	5.8×10^−7^	0.08	25738	−0.05 (0.02)	0.007
rs12148488	15	73169595	*SCAMP5,PPCDC*	3	T	0.50	47341	−0.07 (0.01)	5.9×10^−7^	0.43	25738	−0.06 (0.02)	0.001

Chr, chromosome; EA, effect allele; EAF, effect allele frequency; SE standard error.

*Number of significant SNPs in LD (r^2^ >0.5) and/or located <250 kb from index SNP according to HapMap.

**P value for between study heterogeneity.

Based on the well-established biological link between smoking and *AHR* [8], and *CYP1A2* [9] and caffeine consumption behavior [2], we explored the role of cigarette smoking (Table 3). Compared to our primary model that adjusted for smoking, a model not adjusted for smoking yielded slightly attenuated associations and when restricting analyses to ‘never smokers’ similar regression coefficients were observed as for the complete study population. These findings suggest that smoking is unlikely the cause of the associations observed in our GWAS of caffeine intake.

**Table 3:** Genome-wide meta-analysis of caffeine consumption (*P*<10^−6^): Smoking effects.

Index SNP	Chr	EA	Not Adjusted for Smoking				Never Smokers				Current Smokers			
			N	β	*P*	*P*_het_ *	N	β	*P*	*P*_het_ *	N	β	*P*	*P*_het_ *
rs4410790	7	T	36150	−0.15	8.2×10^−18^	0.18	16809	−0.19	1.8×10^−14^	0.09	5058	−0.10	0.02	0.96
rs2470893	15	T	47612	0.12	5.0×10^−13^	0.70	21413	0.13	3.0×10^−8^	0.19	7466	0.06	0.16	0.56
rs2472304	15	A	47596	0.07	2.4×10^−6^	0.15	21410	0.07	0.0019	0.03	7464	0.03	0.36	0.47
rs6495122	15	A	47612	−0.07	5.2×10^−6^	0.24	21413	−0.07	0.0011	0.03	7466	−0.01	0.75	0.38
rs12148488	15	T	47612	−0.07	1.9×10^−6^	0.63	21413	−0.08	0.0001	0.07	7466	−0.002	0.97	0.27

Chr, chromosome; EA, effect allele;

*P value for between study heterogeneity.

We further conducted 21 candidate gene analyses and found significant gene-based associations (Bonferroni corrected for the total number of human genes) between *CYP2C9* (*P* = 0.023), and *ADORA2A* (*P* = 0.011) and caffeine intake in addition to *CYP1A2* and *AHR* (Table 4).

**Table 4:** Candidate gene-based association results.*

Chr	Gene	#SNPs	#simulations	start position	stop position	Gene-based *P*
1	*ADORA3*	43	1000	111827492	111908120	0.69
1	*FMO3*	26	1000	169326659	169353583	0.17
1	*ADORA1*	43	1000	201363458	201403156	0.13
2	*XDH*	47	1000	31410691	31491115	0.22
5	*DRD1*	33	100000	174800280	174803769	0.10
7	*AHR*	18	1000000	17304831	17352299	<1×10^−6^
7	*CYP3A4*	11	1000	99192539	99219744	0.56
7	*CYP3A43*	3	1000	99263571	99302109	0.58
8	*NAT1*	3	1000	18111894	18125100	0.52
8	*NAT2*	32	1000	18293034	18303003	0.62
10	*CYP2C9*	23	100000	96688404	96739138	0.023
10	*CYP2C8*	20	100000	96786518	96819244	0.05
10	*CYP2E1*	16	1000	135190856	135202610	0.23
11	*DRD2*	34	100000	112785526	112851211	0.077
12	*TAS2R7*	4	1000	10845397	10846493	0.96
12	*TAS2R14*	1	1000	10982119	10983073	0.72
15	*CYP1A2*	11	1000000	72828236	72835994	<1×10^−6^
17	*ADORA2B*	15	1000	15788955	15819935	0.30
17	*PPP1R1B*	19	1000	35036704	35046404	0.74
19	*CYP2A6*	45	1000	46041282	46048192	0.43
19	*CYP2A7*	28	1000	46073183	46080497	0.60
22	*COMT*	41	1000	18309308	18336530	0.27
22	*ADORA2A*	8	100000	23153529	23168325	0.011

*Gene-based analyses were performed using VEGAS [37]. See Materials and Methods for details.

## Discussion

In the first GWAS of caffeine intake in a total of 47,341 individuals from five U.S. studies, loci at 15q24 and 7p21 achieved genome-wide significance. *CYP1A2* at 15q24 and *AHR* at 7p21 are attractive candidate genes for caffeine intake. At plasma concentrations typical of humans (<100 µM), caffeine is predominantly (∼95% of a dose) metabolized by CYP1A2 via N^1^-, N^3^-, and N^7^-demethylation to its three dimethylxanthines, namely, theobromine, paraxanthine, and theophylline, respectively [10]. CYP1A2 expression and activity vary 10- to 60-fold between individuals [11]. Human *CYP1A2* is located immediately adjacent to *CYP1A1* in reverse orientation and the two genes share a common 5′-flanking region [12]. At least 15 *AHR* response elements (AHRE) reside in this bidirectional promoter region and rs2470893 is located in AHRE6 (originally reported as AHRE5[7]) which correlates with transcriptional activation of both *CYP1A1* and *CYP1A2* [6], [7]. *CYP1A1* expression in the liver (the target tissue for caffeine metabolism) is low and there is little evidence that this enzyme contributes to caffeine metabolism. This contrasts with the tissue specific expression of *CYP1A2* in the liver, which suggests further evidence supporting its role in caffeine metabolism. The observation that a stronger association exists for SNPs upstream of the gene suggests that variation in *CYP1A2* gene expression probably affects caffeine intake. The protein product of *AHR*, AhR, is a ligand–activated transcription factor that, upon binding, partners with ARNT and translocates to the nucleus where it regulates the expression of a number of genes including *CYP1A1* and *CYP1A2*. There is marked variation in AhR binding affinity across populations, but so far no polymorphisms have been identified that account for this variation [13]. The most studied SNP, rs2066853 (R554K), is located in exon 10, a region of *AHR* that encodes the transactivation domain[13]. Although this SNP was associated with caffeine in the current study (*P* = 0.0004), our strongest signal mapped upstream of *AHR*, suggesting variation in *AHR* expression has a key role in propensity to consume caffeine. An interaction between CYP1A2 and AHR could be biologically plausible; however, we did not find any evidence supporting statistical interaction between the top two loci (data not shown).

Human and animal candidate gene studies for caffeine intake and related traits have focused on various other genes linked to caffeine's metabolism and targets of action. In our candidate gene analyses, we observed significant gene-based associations between *CYP2C9* and *ADORA2A* and caffeine intake in addition to *CYP1A2* and *AHR*. CYP2C9 catalyzes the N^7^-demethylation and C^8^-hydroxylation of caffeine to theophylline and 1,3,7-trimethyluric acid (a minor metabolite), respectively; but its role relative to CYP1A2 is generally small[10]. In amounts typically consumed from dietary sources, caffeine antagonizes the actions of adenosine at the adenosine A_2A_ receptor (*ADORA2A*) [2], which plays an important role in the stimulating and reinforcing properties of caffeine [14], [15]. Polymorphisms of *ADORA2A* have been previously implicated in caffeine-induced anxiety as well as habitual caffeine intake[16], [17].

All studies contributing to our GWAS of caffeine intake were US-based. Consistent with the adult caffeine consumption pattern of this country, coffee contributed to well over 80% of caffeine intake. Previous studies suggest that some of the heritability underling specific caffeine sources (i.e. coffee and tea) may be distinct in relation to total caffeine intake [18]. To evaluate the robustness of findings, we conducted an additional GWAS analysis using caffeinated coffee intake as the outcome variable yielding the same strong signals (rs4410790: 1.4×10^−29^, rs2470893: 3.6×10^−19^).

Imprecision in phenotypic assessment and differences across studies could have limited the scope of our discovery. Although dietary intake obtained by FFQ is subject to misclassification, validation studies in subsamples of the included studies indicated that the consumption of caffeine-containing beverages is assessed with good accuracy [19], [20], [21]. The cubic root transformation we applied to reported caffeine intakes, however, limits interpretation of the effect estimates. The crude weighted mean difference in caffeine intake between homozygote genotypes was 44 mg/d for rs4410790 and 38 mg/d for rs2470893 (Table S3 and S4). The two SNPs together, however, explained between 0.06 and 0.72% of the total variation in caffeine intake across studies suggesting additional variants remain to be discovered [22]. Finally, our GWAS assumed an additive genetic model and based on study-level results (Figure 1 and Figure 2) potential non-linear effects will require confirmation in future studies.

Caffeine intake has been associated with pleotropic physiologic effects in relation to both detrimental and beneficial health outcomes [23]. Our current study provides insights into the primary pathways underlying caffeine intake. Knowledge of the genetic determinants of caffeine intake may provide insight into underlying mechanisms and may provide ways to study the potential health effects of caffeine more comprehensively by using genetic determinants as instrumental variables for caffeine intake or by taking into consideration caffeine-gene interactions. With the exception of nicotine dependency and the associated nicotinic receptor, genes that influence traits associated with dependency have been difficult to identify. The association of caffeine consumption with genes involved in metabolism or its regulation (CYP1A2 and AhR, respectively) illustrates that it is feasible to use GWAS to identify genetic determinants of other behavioral traits that are assessed with lower accuracy. We also recognize that the identified variants could influence regulation of their genomic elements distant from the known, high profile, neighboring candidate genes. In conclusion, we identified two loci related to caffeine consumption that will be worthy of further investigation with regard to both beneficial and toxic effects of caffeine as well as the extensive group of carcinogens, drugs, and xenobiotics also metabolized through action of the regulation of the gene products of *CYP1A2* and *AHR*.

## Material and Methods

### Ethics Statement

This study was conducted according to the principles expressed in the Declaration of Helsinki. All participants in the contributing studies gave written informed consent including consent for genetic analyses. Local institutional review boards approved study protocols.

### Study Populations

We conducted a meta-analysis of 47,341 individuals of European descent, sourced from Atherosclerosis Risk in Communities **(**ARIC, N = 8,976), the Prostate, Lung, Colorectal, and Ovarian Cancer Screening Trial (PLCO, N = 4,942), the Nurses' Health Study (NHS, N = 6,774), the Health Professionals Follow-Up Study (HPFS, N = 4,023), and the Women's Genome Health Study (WGHS, N = 22,658) to identify novel loci associated with habitual caffeine consumption. Study population descriptions and genotyping quality control for data generated with either the Affymetrix 6.0 or the Illumina Infinium arrays (HumanHap300, 550 or 610 arrays) are provided in Text S1 and Table S5 and S6.

### Caffeine Intake Assessment

In the NHS, every 2 to 4 years of follow-up diet was assessed using a validated semi-quantitative food frequency questionnaire (FFQ) [24]. For the present analysis, we included the participants' mean caffeine intakes of the 1984 (first year in which caffeinated and decaffeinated coffee were differentiated) and 1986 FFQs. The following caffeine-containing foods and beverages were included in the FFQ: coffee with caffeine, tea, cola and other carbonated beverages with caffeine, and chocolate. For each item, participants were asked how often, on average, they had consumed a specified amount of each beverage or food over the past year. The participants could choose from nine frequency categories (never, 1–3 per month, 1 per week, 2–4 per week, 5–6 per week, 1 per day, 2–3 per day, 4–5 per day and 6 or more per day). Intakes of nutrients and caffeine were calculated using US Department of Agriculture food composition sources. In these calculations, we assumed that the content of caffeine was 137 mg per cup of coffee, 47 mg per cup of tea, 46 mg per can or bottle of cola or other caffeinated carbonated beverage, and 7 mg per 1 oz serving of chocolate candy. We assessed the total intake of caffeine by summing the caffeine content for the specified amount of each food multiplied by a weight proportional to the frequency of its use. In a validation among a subsample of this cohort, we obtained high correlations between intake of caffeinated coffee and other caffeinated beverages from the FFQ and four 1-week diet records (coffee, r = 0.78; tea, r = 0.93; and caffeinated sodas, r = 0.85)[21].

In the WGHS, caffeine intake was assessed at baseline (1991) using the same FFQ and caffeine algorithm as the NHS [25].

HPFS participants have been followed with repeated FFQs every 4 years. Caffeine-intake was assessed by the same methods as described above for the NHS cohort. In a validation study in a subsample of participants, we obtained high correlations between consumption of coffee and other caffeinated beverages estimated from the FFQ and consumption estimated from repeated 1-wk diet records (coffee: r = 0.83; tea: r = 0.62; low-calorie caffeinated sodas: r = 0.67; and regular caffeinated sodas: r = 0.56)[21]. For the present analysis, we included the participants mean caffeine intakes of the 1986 (baseline) and 1990 FFQs.

In the ARIC study, caffeine consumption was quantified at the baseline (1987–1989) examination from an interview-administered 66-item semi-quantitative FFQ[19], [20]. The Harvard Nutrition Database was used to assign caffeine (and nutrient) content to each of the food and beverage line items. Line items quantifying consumption of caffeine-containing beverages included sodas (regular and diet), coffee, and tea. The frequency of consumption of each of these items was multiplied by their caffeine content and summed across all beverages to obtain a total caffeine intake value.

Caffeine intake in the PLCO trial was assessed at the randomization phase (between 1992–2001) using responses from a FFQ developed at the National Cancer Institute called the Diet History Questionnaire (DHQ). The DHQ was previously validated against four 24 hour dietary recalls [26] and asks about consumption frequency of 124 food items over the past 12 months, including the primary sources of caffeine: coffee, tea, and soft drinks. For soft drinks, participants selected among 10 possible frequency response categories from “never” to “6+ times per day,” with three possible portion size response categories: <12 ounces or <1 can or bottle; 12–16 ounces or 1 can or bottle; or >16 ounces or >1 can or bottle. Frequency and portion size for coffee and tea were queried together as cups per unit time ranging from “none” to “6 or more cups per day.” For all three of the above beverages, participants were asked the proportion of the time each were consumed in decaffeinated form (almost never or never, about ¼ of the time, about ½ the time, about ¾ of the time, almost always or always). From these responses daily consumption of caffeine was computed taking into account the caffeine content, portion size, and frequency of intake. Caffeine estimates were derived from two 24-hour dietary recalls administered in the 1994-96 Continuing Survey of Food Intake by Individuals (CSFII)[27], a nationally representative survey conducted during the period when the DHQ was being administered. Individual foods/beverages reported on the recalls were placed in food groups consistent with items on the DHQ and weighted mean nutrient values based on survey data were derived for adults stratified by sex using methods previously described [28].

### Imputation

Each study used either MACH [29] (ARIC, NHS, HPFS, WGHS) or IMPUTE [30] (PLCO) to impute up to ∼2.5 million autosomal SNPs with NCBI build 36 of Phase II HapMap CEU data (release 22) as the reference panel. Genotypes were imputed for SNPs not present in the genome-wide arrays or for those where genotyping had failed to meet the quality control criteria. Imputation results are summarized as an “allele dosage” (a fractional value between 0 and 2), defined as the expected number of copies of the minor allele at that SNP.

### Phenotype Harmonization and Model Selection

The algorithm used for the calculation of caffeine intake was study-specific to allow for differences in questionnaires and consumption habits in different study populations. Raw caffeine-intake measures were skewed across studies and after exploring a variety of transformation options, we found that a cubic-root transformation was very close to the most optimal transformation identified by the Box-Cox procedure and was used to ensure normality of the residuals. Our final models were also adjusted for age (continuous), sex, case-control status (if applicable), study-site (if applicable), smoking status (never, former, and current: 2 categories), and study specific eigenvectors (see Table S5 for study-specific models). Adjustment for smoking status was appropriate given the strong correlation between smoking and caffeine intake that might impede our ability to uncover caffeine-specific loci. Each study collected information on smoking status at the time FFQ were administered. A flexible modeling approach was used to accommodate the different methods by which smoking was collected across studies, but all included never, former and two categories of current smokers. Further adjustments for body-mass-index did not change results appreciably.

### Study-Level GWAS

Each study performed genome-wide association testing for normalized caffeine-intake across ∼2.5 million SNPs, based on linear regression under an additive genetic model. Analyses were adjusted for additional covariates as described above and further detailed in Table S5. Imputed data (expressed as allele dosage) were examined using ProbABEL[31] or R (scripts developed in-house). The genomic inflation factor λ for each study as well as the meta-analysis was estimated from the median χ^2^ statistic.

### Meta-Analysis

Meta-analysis was conducted using a fixed effects model and inverse-variance weighting as implemented in METAL (see URLs in Text S1). The software also calculates the genomic control parameter and adjusts each study's standard errors. Fixed effects analyses are regarded as the most efficient method for discovery in the GWAS setting [32]. Heterogeneity across studies was investigated using the *I^2^* statistic[33]. We applied stringent quality filters to imputed SNPs prior to meta-analysis; removing those with <0.02 MAF and/or with low imputation quality scores. The latter was defined as Rsq≤0.80 for SNPs imputed with MACH and proper_info≤0.7 for SNPs imputed with IMPUTE. X and Y chromosome, pseudosomal and mitochondrial SNPs were not included for the present analysis. We retained only SNP-phenotype associations that were based on results from at least 2 of the 10 participating studies and if greater than 50% of the samples contributing to the results were genotyped. Additional checks for experimental biases were implemented for notable associations including manual inspection of SNP (if imputed, an assayed SNP in high LD) cluster plots, and evaluation of HWE, and comparison of study MAFs to the HapMap CEPH panel. We considered P-values <5×10^−8^ to indicate genome-wide significance [34].

### Candidate Gene–Based Analyses

We examined 515 SNPs in 23 genes (±50 kb) either previously studied or members of the key biological pathway: ‘Caffeine metabolism’ (KEGG [35], supplemented with candidates from[10], [36]) for association with caffeine consumption in our GWA meta-analysis sample. SNPs mapping to *TAS2R10*, *43* and *46*, implicated in the oral detection of caffeine, did not pass our stringent QC criteria and thus were not included. Gene-based analyses were performed using VEGAS [37]. The software applies a test that incorporates information from a set of markers within a gene (or region) and accounts for LD between markers by using simulations from the multivariate normal distribution. The number of simulations per gene is determined adaptively. In the first stage, 1000 simulations are performed. If the resulting empirical *P* value is less than 0.1, 10000 simulations are performed. If the empirical *P* value from 10000 simulations is less than 0.0001, the program will perform 1000000 simulations. At each stage, the simulations are mutually exclusive. For computational reasons, if the empirical P value is 0, then no more simulations will be performed. An empirical *P* value of 0 from 1000000 simulations can be interpreted as *P*<10 E-6, which exceeds a Bonferroni-corrected threshold of *P*<2.8E-6 [∼0.05/17,787 (number of autosomal genes)].

## Supporting Information

Figure S1 QQ plots for study-level GWAS of caffeine consumption. Results for genotyped and imputed SNPs denoted by red and blue points, respectively.
(TIFF)

Figure S2 Regional association plots of the two caffeine-associated loci. SNPs are plotted with their meta-analysis P-values (as -log10 values) as a function of genomic position (NCBI Build 36). In each panel, the index association SNP is represented by a diamond. Estimated recombination rates (taken from HapMap CEU) are plotted to reflect the local LD structure. SNP color indicates LD with the index SNP according to a scale from r^2^ = 0 to r^2^ = 1 based on pairwise r^2^ values from HapMap CEU. Plots were created using LocusZoom (see URLs).
(TIFF)

Table S1 Genome-wide meta-analysis of caffeine consumption: All SNPs *P*<10^−4^.
(DOCX)

Table S2 Genome-wide meta-analysis of caffeine consumption (*P*<10^−6^): Gender and study effects.
(DOCX)

Table S3 Mean caffeine intakes (mg/d) by rs4410790 genotype.
(DOCX)

Table S4 Mean caffeine intakes (mg/d) by rs2470893 genotype.
(DOCX)

Table S5 Study-specific genotyping, imputation and statistical analysis.
(DOCX)

Table S6 Sample quality control.
(DOCX)

Text S1 Study population descriptions and URLS.
(DOC)

## References

[1] Frary CD, Johnson RK, Wang MQ (2005). Food sources and intakes of caffeine in the diets of persons in the United States.. J Am Diet Assoc.

[2] Fredholm BB, Battig K, Holmen J, Nehlig A, Zvartau EE (1999). Actions of caffeine in the brain with special reference to factors that contribute to its widespread use.. Pharmacol Rev.

[3] Yang A, Palmer AA, de Wit H (2010). Genetics of caffeine consumption and responses to caffeine.. Psychopharmacology (Berl).

[4] Ferre S (2008). An update on the mechanisms of the psychostimulant effects of caffeine.. J Neurochem.

[5] Tobacco and Genetics Consortium (2010). Genome-wide meta-analyses identify multiple loci associated with smoking behavior.. Nat Genet.

[6] Jorge-Nebert LF, Jiang Z, Chakraborty R, Watson J, Jin L (2010). Analysis of human CYP1A1 and CYP1A2 genes and their shared bidirectional promoter in eight world populations.. Hum Mutat.

[7] Ueda R, Iketaki H, Nagata K, Kimura S, Gonzalez FJ (2006). A common regulatory region functions bidirectionally in transcriptional activation of the human CYP1A1 and CYP1A2 genes.. Mol Pharmacol.

[8] Denison MS, Nagy SR (2003). Activation of the aryl hydrocarbon receptor by structurally diverse exogenous and endogenous chemicals.. Annu Rev Pharmacol Toxicol.

[9] Zhou SF, Wang B, Yang LP, Liu JP (2010). Structure, function, regulation and polymorphism and the clinical significance of human cytochrome P450 1A2.. Drug Metab Rev.

[10] Kot M, Daniel WA (2008). The relative contribution of human cytochrome P450 isoforms to the four caffeine oxidation pathways: an in vitro comparative study with cDNA-expressed P450s including CYP2C isoforms.. Biochem Pharmacol.

[11] Gunes A, Dahl ML (2008). Variation in CYP1A2 activity and its clinical implications: influence of environmental factors and genetic polymorphisms.. Pharmacogenomics.

[12] Corchero J, Pimprale S, Kimura S, Gonzalez FJ (2001). Organization of the CYP1A cluster on human chromosome 15: implications for gene regulation.. Pharmacogenetics.

[13] Harper PA, Wong JY, Lam MS, Okey AB (2002). Polymorphisms in the human AH receptor.. Chem Biol Interact.

[14] Huang ZL, Qu WM, Eguchi N, Chen JF, Schwarzschild MA (2005). Adenosine A2A, but not A1, receptors mediate the arousal effect of caffeine.. Nat Neurosci.

[15] Ledent C, Vaugeois JM, Schiffmann SN, Pedrazzini T, El Yacoubi M (1997). Aggressiveness, hypoalgesia and high blood pressure in mice lacking the adenosine A2a receptor.. Nature.

[16] Rogers PJ, Hohoff C, Heatherley SV, Mullings EL, Maxfield PJ (2010). Association of the anxiogenic and alerting effects of caffeine with ADORA2A and ADORA1 polymorphisms and habitual level of caffeine consumption.. Neuropsychopharmacology.

[17] Cornelis MC, El-Sohemy A, Campos H (2007). Genetic polymorphism of the adenosine A2A receptor is associated with habitual caffeine consumption.. Am J Clin Nutr.

[18] Luciano M, Kirk KM, Heath AC, Martin NG (2005). The genetics of tea and coffee drinking and preference for source of caffeine in a large community sample of Australian twins.. Addiction.

[19] Willett WC, Sampson L, Stampfer MJ, Rosner B, Bain C (1985). Reproducibility and validity of a semiquantitative food frequency questionnaire.. Am J Epidemiol.

[20] Stevens J, Metcalf P, Dennis B, Tell G, Shimakawa T (1996). Reliability of a food frequency questionnaire by ethnicity, gender, age and education.. Nutrition Research.

[21] Feskanich D, Rimm EB, Giovannucci EL, Colditz GA, Stampfer MJ (1993). Reproducibility and validity of food intake measurements from a semiquantitative food frequency questionnaire.. J Am Diet Assoc.

[22] Park JH, Wacholder S, Gail MH, Peters U, Jacobs KB (2010). Estimation of effect size distribution from genome-wide association studies and implications for future discoveries.. Nat Genet.

[23] Higdon JV, Frei B (2006). Coffee and health: a review of recent human research.. Crit Rev Food Sci Nutr.

[24] Willett WC (1998). Nutritional Epidemiology..

[25] Ridker PM, Chasman DI, Zee RY, Parker A, Rose L (2008). Rationale, design, and methodology of the Women's Genome Health Study: a genome-wide association study of more than 25,000 initially healthy american women.. Clin Chem.

[26] Subar AF, Thompson FE, Kipnis V, Midthune D, Hurwitz P (2001). Comparative validation of the Block, Willett, and National Cancer Institute food frequency questionnaires: the Eating at America's Table Study.. Am J Epidemiol.

[27] Tippett K, Cypel Y (1997). Design and Operation: The Continuing Survey of Food Intakes by Individuals and the Diet and Health Knowledge Survey, 1994-96..

[28] Subar AF, Midthune D, Kulldorff M, Brown CC, Thompson FE (2000). Evaluation of alternative approaches to assign nutrient values to food groups in food frequency questionnaires.. Am J Epidemiol.

[29] Li Y, Abecasis GR (2006). Mach 1.0: Rapid haplotype reconstruction and missing genotype inference.. Am J Hum Genet.

[30] Marchini J, Howie B, Myers S, McVean G, Donnelly P (2007). A new multipoint method for genome-wide association studies by imputation of genotypes.. Nat Genet.

[31] Aulchenko YS, Struchalin MV, van Duijn CM (2010). ProbABEL package for genome-wide association analysis of imputed data.. BMC Bioinformatics.

[32] Pereira TV, Patsopoulos NA, Salanti G, Ioannidis JP (2009). Discovery properties of genome-wide association signals from cumulatively combined data sets.. Am J Epidemiol.

[33] Ioannidis JP, Patsopoulos NA, Evangelou E (2007). Heterogeneity in meta-analyses of genome-wide association investigations.. PLoS ONE.

[34] Pe'er I, Yelensky R, Altshuler D, Daly MJ (2008). Estimation of the multiple testing burden for genomewide association studies of nearly all common variants.. Genet Epidemiol.

[35] Kanehisa M, Goto S (2000). KEGG: kyoto encyclopedia of genes and genomes.. Nucleic Acids Res.

[36] Meyerhof W, Batram C, Kuhn C, Brockhoff A, Chudoba E (2010). The molecular receptive ranges of human TAS2R bitter taste receptors.. Chem Senses.

[37] Liu JZ, McRae AF, Nyholt DR, Medland SE, Wray NR (2010). A versatile gene-based test for genome-wide association studies.. Am J Hum Genet.

