# Supplementary figures and images for: Genome-Wide Meta-Analysis Identifies Regions on 7p21 (AHR) and 15q24 (CYP1A2) As Determinants of Habitual Caffeine Consumption
Source: PLoS Genet. 2011 Apr 7;7(4):e1002033. doi: 10.1371/journal.pgen.1002033 (PMC3071630; doi:10.1371/journal.pgen.1002033)

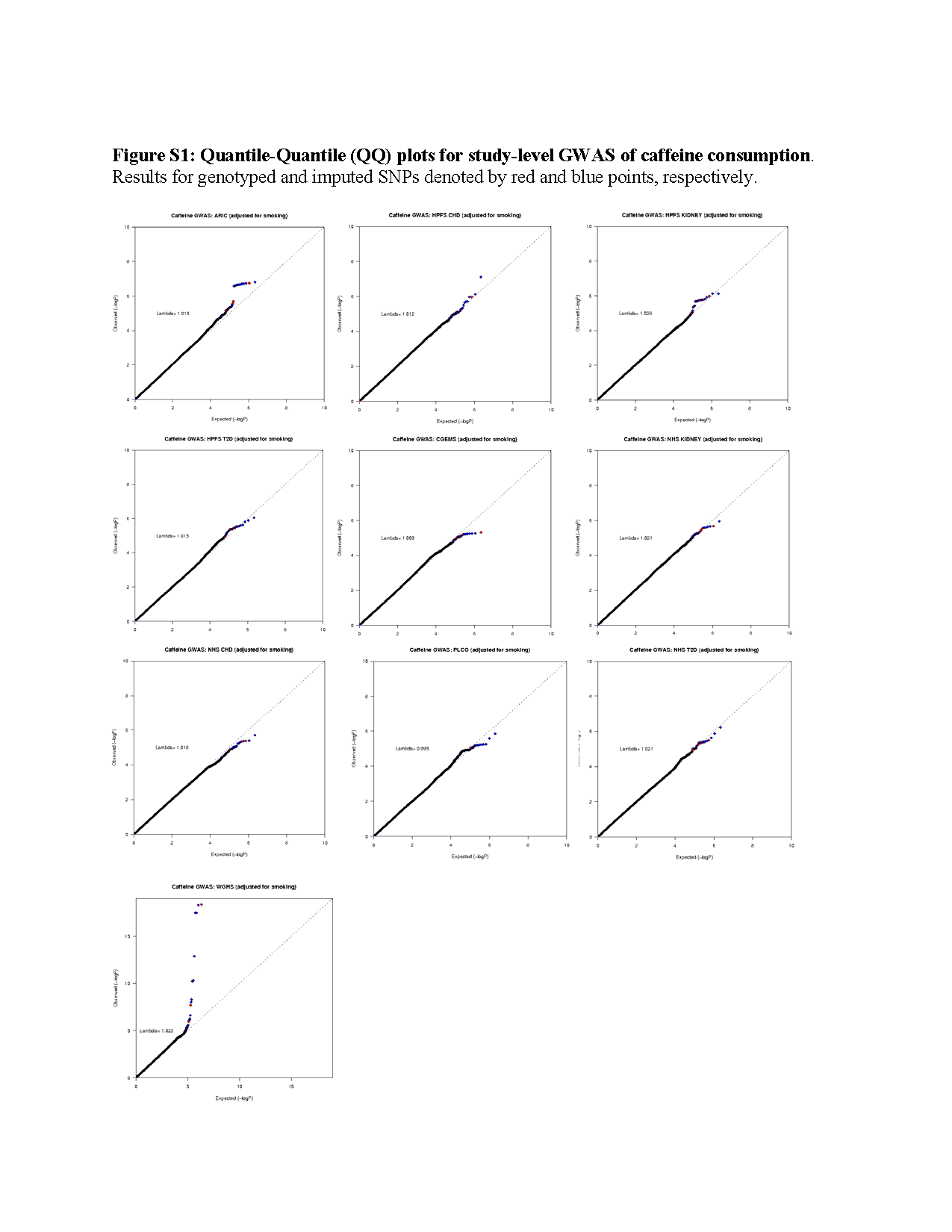

Supplement: Figure S1 — QQ plots for study-level GWAS of caffeine consumption. Results for genotyped and imputed SNPs denoted by red and blue points, respectively. (TIFF) [file pgen.1002033.s001.tiff]

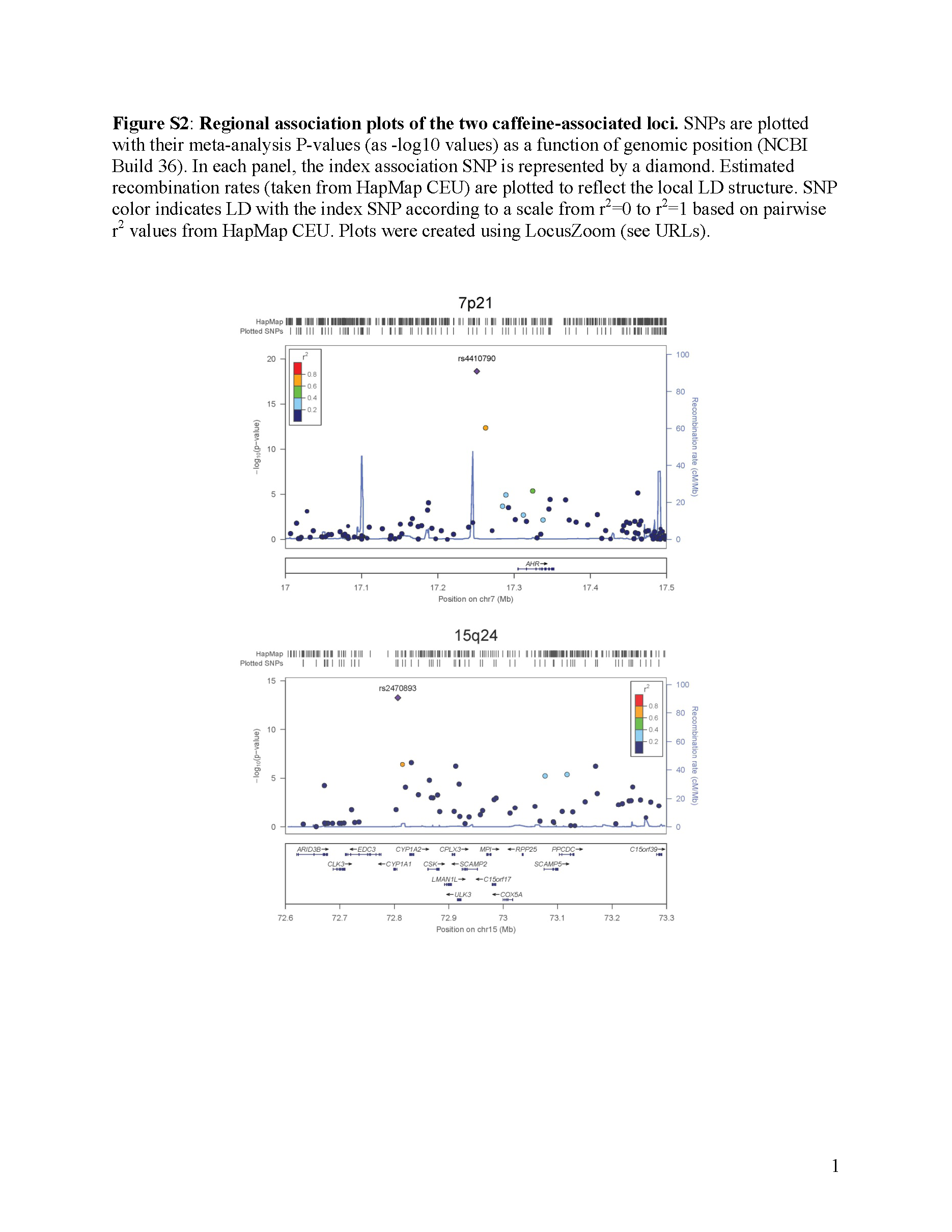

Supplement: Figure S2 — Regional association plots of the two caffeine-associated loci. SNPs are plotted with their meta-analysis P-values (as -log10 values) as a function of genomic position (NCBI Build 36). In each panel, the index association SNP is represented by a diamond. Estimated recombination rates (taken from HapMap CEU) are plotted to reflect the local LD structure. SNP color indicates LD with the index SNP according to a scale from r2 = 0 to r2 = 1 based on pairwise r2 values from HapMap CEU. Plots were created using LocusZoom (see URLs). (TIFF) [file pgen.1002033.s002.tiff]
