# Supplementary material for: Genome-Wide Meta-Analysis Identifies Regions on 7p21 (AHR) and 15q24 (CYP1A2) As Determinants of Habitual Caffeine Consumption
Source: PLoS Genet. 2011 Apr 7;7(4):e1002033. doi: 10.1371/journal.pgen.1002033 (PMC3071630; doi:10.1371/journal.pgen.1002033)
Supplement: Table S1 — Genome-wide meta-analysis of caffeine consumption: All SNPs P<10−4. (DOCX) [file pgen.1002033.s003.docx]

**Table S1. Genome-wide meta-analysis of caffeine consumption: All SNPs *P*<10^-4^**

| SNP | Chr | Position | EA | EAF | N | β | SE | *P* | *P*_het_* |
| --- | --- | --- | --- | --- | --- | --- | --- | --- | --- |
| rs4410790  rs2470893  rs17137472  rs2472304  rs2472297  rs6495122  rs12148488  rs2240466  rs714052  rs4886417  rs1476080  rs7777102  rs8014204  rs2725236  rs16966142  rs3812945  rs7447447  rs1868361  rs2080087  rs3890534  rs4719495  rs11852686  rs10510883  rs1378942  rs8025347  rs908547  rs6753833  rs2185341  rs597837  rs1123848  rs17784583  rs7217677  rs883828  rs7338982  rs2111705  rs7240747  rs1337953  rs10851872  rs4764936  rs2040623  rs17494917  rs9574455  rs936227  rs7675988  rs11706018  rs7791070  rs13180609  rs10893538  rs12424533  rs4740  rs4941161  rs2821017  rs4954564  rs12898794  rs1043823  rs949845  rs10092214  rs998939  rs3745376  rs2967072  rs1016438  rs12965440  rs953387  rs17475653  rs2278483  rs1045750  rs8040372  rs6545803  rs784600  rs1569951  rs2472299  rs4942023  rs7610023  rs16956362  rs3857915  rs7604754  rs133629  rs11653144  rs1464906  rs9309308  rs1966764  rs13235734  rs702933  rs2787087  rs10495985  rs11597105 | 7  15  7  15  15  15  15  7  7  15  7  7  14  4  16  15  5  7  14  16  7  15  3  15  15  2  2  1  10  2  16  17  17  13  14  18  13  15  12  7  2  13  15  4  3  7  5  11  12  19  18  1  2  15  1  5  8  11  19  5  19  18  2  13  2  19  15  2  1  14  15  13  3  15  8  2  22  17  7  2  15  7  2  13  2  10 | 17251102  72806502  17262597  72831291  72814933  72912698  73169595  72494205  72502805  73117212  17324398  72695953  74392547  89138130  88378534  73076775  104895835  17462194  74434815  88362790  17289208  72502258  62631299  72864420  90660285  113186630  50100653  22598412  21342827  136661499  88554480  24799793  24777972  98408374  74331394  2292770  33728822  72577236  101998198  17347187  50104262  79142737  72919012  126256226  140399083  17367552  104939367  125927580  50854768  4187996  58661467  3510731  136611978  72671500  200112055  171983515  61889433  95054261  7739690  36434134  62282850  34858474  136623640  100100437  24893586  4202069  73237610  60387703  39912140  22677111  72820453  40573611  68206421  28986264  93258807  50083838  47085180  24699352  17187588  24848074  98791838  90268135  65595119  33806957  49946265  6552587 | T  T  T  A  T  A  T  A  A  T  T  A  A  A  T  T  T  T  A  T  T  T  A  A  A  A  A  T  A  T  T  A  A  T  A  T  A  A  T  A  A  A  A  T  T  T  A  A  T  A  T  A  A  T  T  T  A  T  T  A  A  T  A  A  T  T  A  T  T  T  A  A  A  A  A  T  A  T  A  T  T  A  T  A  T  A | 0.3762  0.3089  0.4985  0.6512  0.2391  0.4331  0.504  0.1209  0.8793  0.5594  0.3665  0.8568  0.5273  0.4765  0.0828  0.5425  0.1255  0.1745  0.4702  0.0822  0.1962  0.5141  0.2736  0.6613  0.1677  0.5419  0.4297  0.2965  0.6245  0.3227  0.8598  0.1778  0.1777  0.6188  0.5488  0.4929  0.3463  0.0812  0.1949  0.7866  0.863  0.3348  0.3929  0.094  0.0809  0.782  0.1452  0.0309  0.216  0.287  0.0232  0.349  0.6664  0.6893  0.0938  0.2614  0.4635  0.2004  0.1613  0.9633  0.0887  0.9656  0.666  0.8912  0.2272  0.7117  0.3965  0.251  0.4557  0.503  0.2842  0.2242  0.5837  0.9533  0.1599  0.1033  0.8957  0.3395  0.6646  0.7766  0.1081  0.9431  0.0973  0.4951  0.886  0.1385 | 36013  47341  47321  47325  24673  47341  47341  42398  42398  47341  47339  32702  47331  47340  47331  44960  22634  47339  47340  47337  47337  42400  47340  47341  42397  42400  47341  47334  24670  42400  47332  42396  42396  47339  47340  16657  47339  42398  47338  47326  47339  47339  47339  47340  47340  47339  24620  7716  8003  47341  25738  25738  42400  42400  42398  42400  47340  42399  47340  24673  47339  24661  42400  47338  47341  47341  28873  42400  42397  8009  47336  47339  42399  47341  47315  47341  47331  39264  42397  47339  47340  47326  42400  47339  47341  47330 | -0.149  0.1196  0.1043  0.077  0.1217  -0.0725  -0.071  0.1109  -0.1095  -0.0665  -0.0675  -0.1191  0.065  -0.065  0.118  -0.0685  0.1521  -0.0844  -0.0631  0.1145  -0.079  -0.0633  0.069  0.0649  -0.0851  -0.0629  0.0613  -0.0659  0.0898  0.0682  -0.0869  0.08  0.0799  -0.0613  -0.0599  -0.1041  0.0623  -0.1114  0.0746  0.0716  -0.0853  0.0628  -0.0602  -0.0999  0.1081  0.071  0.1186  -0.3688  0.1829  0.065  0.2457  0.0772  -0.065  0.0641  0.1014  -0.0675  -0.0578  -0.073  -0.0773  -0.2148  0.0991  -0.2251  -0.0637  0.0908  -0.0672  -0.062  -0.0694  -0.0666  0.058  0.1461  -0.0621  -0.0676  0.0581  -0.1335  0.078  0.0921  0.0911  -0.0645  0.0609  0.0669  -0.0897  -0.1251  0.0967  0.0554  -0.0876  -0.0806 | 0.0166  0.0159  0.0144  0.0149  0.024  0.0145  0.0142  0.0228  0.0226  0.0144  0.0147  0.0259  0.0142  0.0143  0.026  0.0151  0.0338  0.0188  0.0142  0.026  0.018  0.0146  0.016  0.015  0.0197  0.0146  0.0143  0.0155  0.0211  0.0162  0.0206  0.019  0.019  0.0146  0.0143  0.0249  0.015  0.0269  0.018  0.0173  0.0207  0.0153  0.0146  0.0243  0.0263  0.0173  0.029  0.0901  0.0448  0.016  0.0603  0.0191  0.0161  0.0159  0.0252  0.0168  0.0145  0.0183  0.0194  0.0539  0.0249  0.0567  0.016  0.0229  0.017  0.0157  0.0176  0.0169  0.0147  0.037  0.0158  0.0172  0.0148  0.0339  0.0198  0.0234  0.0232  0.0164  0.0155  0.017  0.0228  0.0319  0.0247  0.0142  0.0224  0.0207 | 2.36E-19  5.16E-14  4.22E-13  2.53E-07  3.88E-07  5.76E-07  5.94E-07  1.10E-06  1.24E-06  4.09E-06  4.19E-06  4.27E-06  4.86E-06  5.44E-06  5.57E-06  5.72E-06  6.83E-06  6.92E-06  9.18E-06  0.0000105  0.0000115  0.0000146  0.0000153  0.0000156  0.0000161  0.0000169  0.0000187  0.000021  0.0000214  0.0000246  0.0000252  0.0000253  0.0000261  0.000028  0.0000285  0.0000287  0.0000308  0.0000336  0.0000349  0.0000367  0.0000378  0.0000383  0.0000391  0.0000401  0.0000404  0.0000406  0.0000426  0.0000428  0.0000445  0.0000465  0.0000467  0.0000518  0.0000537  0.0000548  0.0000564  0.0000578  0.0000629  0.0000661  0.0000672  0.0000674  0.0000699  0.0000711  0.0000717  0.0000743  0.0000749  0.0000759  0.0000778  0.0000787  0.0000797  0.0000798  0.0000804  0.0000815  0.0000826  0.0000833  0.0000837  0.0000844  0.0000846  0.0000854  0.0000860  0.0000862  0.0000863  0.0000884  0.0000916  0.0000919  0.0000927  0.0000985 | 0.14  0.68  0.24  0.06  0.67  0.084  0.44  0.86  0.86  0.64  0.30  0.19  0.23  0.69  0.14  0.62  0.68  0.77  0.18  0.15  0.77  0.27  0.94  0.054  0.94  0.25  0.29  0.98  0.72  0.63  0.27  0.40  0.40  0.50  0.62  0.58  0.17  0.75  0.55  0.45  0.57  0.71  0.17  0.66  0.18  0.40  0.21  0.27  0.99  0.83  0.59  0.99  0.55  0.62  0.21  0.64  0.61  0.42  0.73  0.10  0.075  0.59  0.62  0.63  0.69  0.76  0.79  0.078  0.89  0.064  0.11  0.99  0.78  0.56  0.81  0.68  0.84  0.14  0.28  0.71  0.86  0.97  0.73  0.31  0.71  0.82 |

Chr, chromosome; EA, effect allele; EAF, effect allele frequency; SE standard error

**P* value for between study heterogeneity
