# Supplementary material for: Genome-Wide Meta-Analysis Identifies Regions on 7p21 (AHR) and 15q24 (CYP1A2) As Determinants of Habitual Caffeine Consumption
Source: PLoS Genet. 2011 Apr 7;7(4):e1002033. doi: 10.1371/journal.pgen.1002033 (PMC3071630; doi:10.1371/journal.pgen.1002033)
Supplement: Table S2 — Genome-wide meta-analysis of caffeine consumption (P<10−6): Gender and study effects. (DOCX) [file pgen.1002033.s004.docx]

**Table S2. Genome-wide meta-analysis of caffeine consumption (*P*<10^-6^): Gender and study effects**

| Index SNP | Chr | EA | Primary Analysis,  All Studies & Subjects | | | | Females | | | | Males | | | | Excluding WGHS | | | |
| --- | --- | --- | --- | --- | --- | --- | --- | --- | --- | --- | --- | --- | --- | --- | --- | --- | --- | --- |
|  |  |  | N | β | *P* | *P*_het_* | N | β | *P* | *P*_het_* | N | β | *P* | *P*_het_* | N | β | *P* | *P*_het_* |
| rs4410790 | 7 | T | 36013 | -0.15 | 2.4×10^-19^ | 0.14 | 27988 | -0.15 | 2.4×10^-16^ | 0.04 | 5420 | -0.16 | 0.0053 | 0.75 | 13355 | -0.09 | 0.0019 | 0.63 |
| rs2470893 | 15 | T | 47341 | 0.12 | 5.2×10^-14^ | 0.68 | 32709 | 0.12 | 9.5×10^-11^ | 0.51 | 12027 | 0.16 | 5.3×10^-5^ | 0.94 | 24683 | 0.12 | 3.5×10^-7^ | 0.58 |
| rs2472304 | 15 | A | 47325 | 0.08 | 2.5×10^-7^ | 0.06 | 32706 | 0.07 | 3.1×10^-5^ | 0.19 | 12014 | 0.12 | 0.00061 | 0.17 | 24667 | 0.09 | 3.1×10^-5^ | 0.05 |
| rs6495122 | 15 | A | 47341 | -0.07 | 5.8×10^-7^ | 0.09 | 32709 | -0.07 | 1.8×10^-5^ | 0.07 | 12027 | -0.10 | 0.0024 | 0.36 | 24683 | -0.08 | 0.00011 | 0.06 |
| rs12148488 | 15 | T | 47341 | -0.07 | 5.9×10^-7^ | 0.43 | 32709 | -0.08 | 2.2×10^-6^ | 0.51 | 12027 | -0.07 | 0.048 | 0.23 | 24683 | -0.07 | 0.00029 | 0.35 |

Chr, chromosome; EA, effect allele

**P* value for between study heterogeneity
