# Supplementary material for: Genome-Wide Meta-Analysis Identifies Regions on 7p21 (AHR) and 15q24 (CYP1A2) As Determinants of Habitual Caffeine Consumption
Source: PLoS Genet. 2011 Apr 7;7(4):e1002033. doi: 10.1371/journal.pgen.1002033 (PMC3071630; doi:10.1371/journal.pgen.1002033)
Supplement: Table S3 — Mean caffeine intakes (mg/d) by rs4410790 genotype. (DOCX) [file pgen.1002033.s005.docx]

**Table S3. Mean caffeine intake (mg/d) by rs4410790 genotype***

| Study | N | Mean intake (mg/d) | | | Difference in mean  intake (mg/d): C/C - T/T |
| --- | --- | --- | --- | --- | --- |
|  |  | C/C | C/T | T/T |  |
| NHS BrCa  NHS CHD  NHS T2D  NHS KS  HPFS CHD  HPFS KS  PLCO  WGHS | 2049  1102  3133  488  1099  543  4941  22658 | 292.6  324.6  291.4  266.0  258.6  247.0  506.6  315.0 | 293.2  305.1  283.3  256.5  238.8  235.0  492.0  295.0 | 247.0  331.4  269.3  286.1  217.9  193.5  443.2  268.0 | 45.6  -6.8  22.1  -20.1  40.7  53.5  63.4  47.0 |
| Total (weighted by N) | | | | | 44.4 |

*****For imputed SNPs, ‘genotype’ corresponds to most probable genotype
