# Supplementary material for: Genome-Wide Meta-Analysis Identifies Regions on 7p21 (AHR) and 15q24 (CYP1A2) As Determinants of Habitual Caffeine Consumption
Source: PLoS Genet. 2011 Apr 7;7(4):e1002033. doi: 10.1371/journal.pgen.1002033 (PMC3071630; doi:10.1371/journal.pgen.1002033)
Supplement: Table S4 — Mean caffeine intakes (mg/d) by rs2470893 genotype. (DOCX) [file pgen.1002033.s006.docx]

**Table S4. Mean caffeine intake (mg/d) by rs2470893 genotype***

| Study | N | Mean intake (mg/d) | | | Difference in mean  intake (mg/d): C/C - T/T |
| --- | --- | --- | --- | --- | --- |
|  |  | C/C | C/T | T/T |  |
| NHS BrCa  NHS CHD  NHS T2D  NHS KS  HPFS CHD  HPFS T2D  HPFS KS  ARIC  PLCO  WGHS | 2049  1102  3135  488  1099  2381  543  8945  4941  22658 | 285.0  307.0  278.0  260.0  231.0  246.0  220.0  319.0  479.0  288.0 | 286.0  323.0  288.0  256.0  249.0  254.0  235.0  337.0  495.0  306.0 | 299.0  345.0  309.0  318.0  297.0  283.0  260.0  372.0  529.0  319.0 | -14.0  -38.0  -31.0  -58.0  -66.0  -37.0  -40.0  -53.0  -50.0  -31.0 |
| Total (weighted by N) | | | | | -38.1 |

*****For imputed SNPs, ‘genotype’ corresponds to most probable genotype
