# Supplementary material for: Genome-Wide Meta-Analysis Identifies Regions on 7p21 (AHR) and 15q24 (CYP1A2) As Determinants of Habitual Caffeine Consumption
Source: PLoS Genet. 2011 Apr 7;7(4):e1002033. doi: 10.1371/journal.pgen.1002033 (PMC3071630; doi:10.1371/journal.pgen.1002033)
Supplement: Table S5 — Study-specific genotyping, imputation and statistical analysis. (DOCX) [file pgen.1002033.s007.docx]

**Table S5. Study-specific genotyping, imputation and statistical analysis**

| **Study** | **Genotyping** | | | | | **Imputation** | | | **Association Analysis** | | | |
| --- | --- | --- | --- | --- | --- | --- | --- | --- | --- | --- | --- | --- |
|  | **Platform** | **Inclusion criteria** | | | **SNPs met**  **QC criteria** | **Software** | **Inclusion criteria** | | **SNPs in**  **meta-analysis** | **λ_GC_** | **Software** | **covariates** |
|  |  | **MAF** | **Call rate** | **P HWE** |  |  | **MAF** | **Imputation quality** |  |  |  |  |
| ARIC | Affymetrix 6.0 | none | ≥95% | ≥10^-6^ | 839,048 | MACH | >2% | Rsq ≥0.8 | 2198440 | 1.02 | ProbABEL | age, sex, study center, smoking (never, former, ≤20 cig/d, >20 cig/d), top 10 eigenvectors |
| PLCO | Illumina 550k  Illumina 610Q | Minimum of 10 observations/ allele | ≥95% | -- | 515, 922 | IMPUTE | >2% | Proper_info≥0.7 | 1990609 | 1.00 | R (in-house scripts) | age, sex, case-control status, smoking (never, former, ≤20 cig/d, >20 cig/d), top 2 eigenvectors |
| NHS T2D | Affymetrix 6.0 | ≥2% | >98% | ≥10^-4^ | 704,409 | MACH | >2% | Rsq ≥0.8 | 2186671 | 1.02 | ProbABEL | age, T2D case-control status, smoking (never, former, <15 cig/d, ≥15 cig/d), top 3 eigenvectors |
| NHS CHD | Affymetrix 6.0 | ≥2% | >98% | ≥10^-4^ | 721,316 | MACH | >2% | Rsq ≥0.8 | 2196994 | 1.02 | ProbABEL | age, CHD case-control status, smoking (never, former, <15 cig/d, ≥15 cig/d), top 3 eigenvectors |
| NHS KS | Illumina 610Q | ≥1% | ≥95% | ≥10^-5^ | 546,344 | MACH | >2% | Rsq ≥0.8 | 2244671 | 1.02 | ProbABEL | age, KS case-control status, smoking (never, former, <15 cig/d, ≥15 cig/d), top 4 eigenvectors |
| NHS BrC | Illumina 550k | ≥1% | ≥90% | - | 528,173 | MACH | >2% | Rsq ≥0.8 | 2244803 | 1.01 | ProbABEL | age, BrC case-control status, smoking (never, former, <15 cig/d, ≥15 cig/d), top 4 eigenvectors |
| HPFS T2D | Affymetrix 6.0 | ≥2% | >98% | ≥10^-4^ | 706,040 | MACH | >2% | Rsq ≥0.8 | 2186837 | 1.02 | ProbABEL | age, T2D case-control status, smoking (never, former, <15 cig/d, ≥15 cig/d), top 4 eigenvectors |
| HPFS CHD | Affymetrix 6.0 | ≥2% | >98% | ≥10^-4^ | 724,881 | MACH | >2% | Rsq ≥0.8 | 2197510 | 1.01 | ProbABEL | age, CHD case-control status, smoking (never, former, <15 cig/d, ≥15 cig/d), top 3 eigenvectors |
| HPFS KS | Illumina 610Q | ≥1% | ≥95% | ≥10^-5^ | 546,344 | MACH | >2% | Rsq ≥0.8 | 2244671 | 1.03 | ProbABEL | age, KS case-control status, smoking (never, former, <15 cig/d, ≥15 cig/d), top 4 eigenvectors |
| WGHS | Illumina HumanHap300 Duo+ (some iSelect) | ≥1% | ≥90% | ≥10^-6^ | 335,603  (includes 32,521 custom content) | MACH | >2% | Rsq ≥0.8 | 2143590 | 1.04 | ProbABEL | age, smoking (never, former, ≤20 cig/d, >20 cig/d), top 5 eigenvectors |
