# Supplementary material for: Genome-Wide Meta-Analysis Identifies Regions on 7p21 (AHR) and 15q24 (CYP1A2) As Determinants of Habitual Caffeine Consumption
Source: PLoS Genet. 2011 Apr 7;7(4):e1002033. doi: 10.1371/journal.pgen.1002033 (PMC3071630; doi:10.1371/journal.pgen.1002033)
Supplement: Table S6 — Sample quality control. (DOCX) [file pgen.1002033.s008.docx]

**Table S6. Sample quality control**

| **Study** | **Sample Quality Control** | | **Sample size included** |
| --- | --- | --- | --- |
|  | **Call rate** | **Other exclusion criteria** |  |
| ARIC | >95% | -no DNA consent  -first-degree relatives  -race discrepancies  - sex/gender phenotype and genotype mismatch  -non-concordance between Affy/Birdsuite genotype and genotype assayed by TaqMan (47 SNPs)  -missing phenotype information | 8,945 |
| PLCO | >98% | -sex discrepancy with genetic data from X-linked markers  -duplicates and first/second degree relatives  -ancestry outliers  -heterozygosity  -autosomal chromosome abberations  -missing phenotype information | 4,942 |
| NHS T2D | >98% | -sex discrepancy with genetic data from X-linked markers  -duplicates and first/second degree relatives  -ancestry outliers  -heterozygosity  -autosomal chromosome abberations  -missing phenotype information | 3,135 |
| NHS CHD | >98% | -sex discrepancy with genetic data from X-linked markers  -duplicates and first/second degree relatives  -ancestry outliers  -heterozygosity  -missing phenotype information | 1,102 |
| NHS KS | ≥95% | -duplicates and first/second degree relatives  -ancestry outliers  -missing phenotype information | 488 |
| NHS BrC | >90% | -duplicates and first/second degree relatives  -ancestry outliers  -missing phenotype information | 2,049 |
| HPFS T2D | >98% | -sex discrepancy with genetic data from X-linked markers  -duplicates and first/second degree relatives  -ancestry outliers  -heterozygosity  -autosomal chromosome abberations  -missing phenotype information | 2,381 |
| HPFS CHD | >98% | -sex discrepancy with genetic data from X-linked markers  -duplicates and first/second degree relatives  -ancestry outliers  -heterozygosity  -missing phenotype information | 1,099 |
| HPFS KS | ≥95% | -duplicates and first/second degree relatives  -ancestry outliers  -missing phenotype information | 543 |
| WGHS | >98% | -ancestry outliers  -missing phenotype information | 22,658 |
