# Supplementary material for: Genome-Wide Meta-Analysis Identifies Regions on 7p21 (AHR) and 15q24 (CYP1A2) As Determinants of Habitual Caffeine Consumption
Source: PLoS Genet. 2011 Apr 7;7(4):e1002033. doi: 10.1371/journal.pgen.1002033 (PMC3071630; doi:10.1371/journal.pgen.1002033)
Supplement: Text S1 — Study population descriptions and URLS. (DOC) [file pgen.1002033.s009.doc]

**Study population descriptions**

*Atherosclerosis Risk in Communities (ARIC)*

The ARIC study is a multi-center prospective investigation of atherosclerotic disease in a predominantly bi-racial population [1]. Men and women aged 45-64 years at baseline were recruited from 4 communities: Forsyth County, North Carolina; Jackson, Mississippi; suburban areas of Minneapolis, Minnesota; and Washington County, Maryland. A total of 15,792 individuals participated in the baseline examination in 1987-1989, with follow-up examinations in approximate 3-year intervals, during 1990-1992, 1993-1995, and 1996-1998. For the present analyses, caffeine consumption was quantified at the baseline examination from an interview-administered 66-item semi-quantitative food frequency questionnaire (FFQ)[2,3].

ARIC Study samples were genotyped using the Affymetrix Genome-Wide Human SNP Array 6.0 (Santa Clara, California). Details regarding genotyping quality control (QC) and assurance have been reported elsewhere [4] and presented in Tables S5 and S6. 9,349 white participants with quality genome-wide scans were considered for the current study. Of these, 8945 participants had data on caffeine and covariates. To control for sub-structure, principal components were generated using the EIGENSTRAT software [5]. A dataset of 8,997 individuals was used to generate eigenvalues for each individual along each of the first 10 principle components (no outlier removal). After the generation of PCs, 134 second-degree relatives were re-included into this dataset and assigned the eigenvalues of the member of the second-degree relative pair that had remained in the analysis. Ten second-degree relatives could not be assigned PCs back because their relatives were excluded for genetic outliers.

*Prostate, Lung, Colorectal, and Ovarian Cancer Screening Trial (PLCO)*

The Prostate, Lung, Colorectal and Ovarian Cancer Screening Trial (PLCO) is a large, randomized controlled trial investigating the efficacy of cancer screening to prevent early death from prostate, lung, colorectal and ovarian cancer^46^. Between 1992 and 2001, approximately 155,000 women and men in ten U.S. cities were enrolled in PLCO and randomized to either a screening or control arm. Eligibility criteria included an age at enrollment between 55 and 74 years and no history of prostate, lung, colon and ovarian cancer, although prior diagnoses of other cancers were acceptable. Individuals were followed up for all cancer diagnoses by annual mailed questionnaire and, additionally for trial disease outcomes, by screening examinations during the first six years of follow-up. Blood specimens were collected annually from screening-arm participants as part of the screening examinations. Buccal cell specimens were collected from control-arm participants. In total, approximately 112,500 participants provided blood or buccal cell specimens. Caucasian subjects included for the current study were selected from both arms of the trial based on availability of a valid baseline questionnaire, FFQ, consent, and GWAS data (N=4,942).

Details regarding genotyping QC and assurance have been reported elsewhere [6] and presented in Tables S5 and S6. STRUCTURE was used to estimate the continental ancestry, with the set of 209 HapMap II founders (59 CEU, 60 YRI, 45 JPT and 45 CHB) as reference populations. Subjects with less than 80% estimated European ancestry were removed.

*Nurses' Health Study (NHS)*

The NHS was established in 1976 when 121,700 female registered nurses aged 30-55 years and residing in 11 large U.S. states completed a mailed questionnaire on medical history and lifestyle characteristics [7]. Every two years, follow-up questionnaires have been sent to update information on exposures and newly diagnosed diseases and every 2 to 4 years diet was assessed using a validated semi-quantitative FFQ [8]. For the present analysis, we included the participants’ mean caffeine intakes of the 1984 (first year in which caffeinated and decaffeinated coffee were differentiated) and 1986 FFQs. Participants for the current study were those with information on caffeine consumption and genome-wide scan data.

Blood was collected from 32,826 NHS members between 1989 and 1990. DNA was extracted from white blood cells using the QIAmp^TM^ (QIAGEN Inc., Chatsworth, CA) blood protocol and all samples were processed in the same laboratory. Genome-wide scans that contribute to this meta-analysis were obtained from 4 independent GWAS of the NHS cohort, initially designed for outcomes of type 2 diabetes (T2D), coronary heart disease (CHD), breast cancer (BrCa) and kidney stone (KS) disease. Both cases and controls were included for analysis. The NHS T2D GWAS is a part of the GENEVA consortium [9]. Controls were defined to be those free of diabetes at the time of diagnosis of the case, and were initially matched on year of birth, month of blood collection, and fasting status, with matched-pairs subsequently broken because not all subjects gave informed consent for the posting of their data on dbGaP. All genotyping was done at the Broad Center for Genotyping and Analysis using the Affymetrix Genome-Wide Human (Affy) 6.0 array and the Birdseed calling algorithm [10]. Details regarding the study design, genotyping quality control (QC) and assurance have been reported elsewhere [4,11]. A total of 3286 unrelated genetically defined White women passing QC were considered for the current study. Of these, 3135 had information on caffeine consumption. The NHS CHD study samples were genotyped at Rosetta/Merck using the Affy 6.0 array and the Birdseed calling algorithm [10]. Controls were randomly selected from participants who provided blood samples and did not experience CHD with two controls for every case. Controls were matched on age, smoking, and month of blood draw. A total of 1146 unrelated genetically defined White women passing QC were considered for the current study. Of these, 1102 had information on caffeine consumption. The NHS BrCa study samples were genotyped using Illumina Human-Hap550 array, as part of the National Center Institute’s Cancer Genetic Markers of Susceptibility (CGEMS) Project [12]. Cases and controls were limited to post-menopausal women, who were not diagnosed with breast cancer during follow up. Controls were postmenopausal women matched with cases by age and post-menopausal hormone use at blood draw. This sample includes 2287 women of genetically defined White ancestry that passed QC procedures described in detail elsewhere [12]. Of these 2287 women, 2049 had caffeine data. The NHS KS samples were genotyped at the Broad Center using the Illumina 610Q array. Participants with a history of kidney stones and randomly selected cases were identified in two cycles from those with no history of cancer (cycles 1 and 2) or cardiovascular disease (cycle 1) who met age eligibility requirements (cycle 1: <66; cycle 2: <76)[13]. This sample includes 504 women of White ancestry that passed QC procedures. 488 of these women had information regarding caffeine intakes.

Although exact QC protocols varied by sample set (Tables S5 and S6), at a minimum DNA samples that did not meet a 90% completion threshold, and SNPs with low call rates (<90%), were dropped. Analyses based on principal components [5] were conducted to assess self-reported race and any self-reported "white" samples that had substantial similarity to non-European reference samples (either the HapMap YRI or CHB+JPT samples) were excluded.

*Health Professionals Follow-up Study (HPFS)*

The HPFS was initiated in 1986 when 51,529 male health professionals between 40 and 75 years of age years and residing in the U.S. completed an FFQ and a questionnaire on lifestyle and medical history. The participants have been followed with repeated questionnaires on lifestyle and health every 2 years and FFQs every 4 years.  For the present analysis, we included the participants mean caffeine intakes of the 1986 and 1990 FFQs. Participants for the current study were those with information on caffeine consumption and genome-wide scan data.

Between 1993 and 1996, a blood sample was requested from all active participants in the HPFS and collected from 18,225 men [14]. DNA was extracted from white blood cells using the QIAmp^TM^ (QIAGEN Inc., Chatsworth, CA) blood protocol and all samples were processed in the same laboratory. Participants for the current study were those with information on caffeine consumption and who also had genome-wide scan data. Genome-wide scans were obtained from 3 independent GWAS of the HPFS cohort, initially designed for outcomes of T2D, CHD and KS disease. Both cases and controls were included for analysis. The HPFS T2D, CHD and KS GWAS followed the same design, genotyping and QC protocols as those described above for the NHS T2D, CHD and KS GWAS, respectively. For HPFS KS, men <71 years of age at cycle 1 or <76 at cycle 2 were eligible for selection. A total of 2484 HPFS T2D, 1146 CHD and 600 KS samples confirmed unrelated and genetically defined White men were considered for the current study. 2381, 1099 and 543 of these, respectively, had information on caffeine consumption. Although exact protocols varied by sample set (Tables S5 and S6), at a minimum DNA samples that did not meet a 95% completion threshold, and SNPs with low call rates (≤95%), were dropped. Analyses based on principal components[5] were conducted to assess self-reported race and any self-reported "white" samples that had substantial similarity to non-European reference samples (either the HapMap YRI or CHB+JPT samples) were excluded.

*Women’s Genome Health Study (WGHS)*

WGHS is a prospective cohort of female healthcare professionals, aged 45 or older at baseline, who provided baseline blood sample and consent for blood based analysis in the Women’s Health Study (WHS), a randomized, placebo controlled trial of aspirin and vitamin E in the primary prevention of cardiovascular disease and cancer. A complete description of the WGHS has been published previously[15]. Caffeine intake was assessed at baseline using the same FFQ as the NHS. Participants for the current study were limited to those with information on caffeine consumption, smoking and who additionally provided a baseline blood sample and for whom complete genome-wide scan data was available.

Genotyping in the WGHS sample was performed using the HumanHap300 Duo ‘‘+ ’’ chips or the combination of the HumanHap300 Duo and iSelect chips (Illumina, San Diego, CA) with the Infinium II protocol. In either case, the custom SNP content was the same; these custom SNPs were chosen without regard to minor allele frequency (MAF) to saturate candidate genes for cardiovascular disease as well as to increase coverage of SNPs with known or suspected biological function, e.g. disease association, non-synonymous changes, substitutions at splice sites, etc. Details regarding genotyping QC and assurance have been reported elsewhere [15] and presented in Tables S5 and S6. A subset of 23,294 individuals were identified with self-reported European ancestry that could be verified on the basis of multidimensional scaling analysis of identity by state using 1443 ancestry informative markers in PLINK v. 1.06.

**URLs.**

R statistical environment, [http://cran.r-project.org/](http://www.google.com/url?q=http%3A%2F%2Fcran.r-project.org%2F&sa=D&sntz=1&usg=AFQjCNEofmii_GUpW_tQP2hCPr3cagpB1Q) ;

PLINK, [http://pngu.mgh.harvard.edu/~purcell/plink/](http://www.google.com/url?q=http%3A%2F%2Fpngu.mgh.harvard.edu%2F%257Epurcell%2Fplink%2F&sa=D&sntz=1&usg=AFQjCNH9nt4JszRY1EhYEFfxZXlCFtHIDQ) ;

MACH 1.0, [http://www.sph.umich.edu/csg/abecasis/mach/](http://www.google.com/url?q=http%3A%2F%2Fwww.sph.umich.edu%2Fcsg%2Fabecasis%2Fmach%2F&sa=D&sntz=1&usg=AFQjCNHGmA8rU6Q8yjtU58NgzRzU-YqKaQ) ;

International HapMap Project, [http://www.hapmap.org/index.html](http://www.google.com/url?q=http%3A%2F%2Fwww.hapmap.org%2Findex.html&sa=D&sntz=1&usg=AFQjCNEcB3-2sMZMA1ASuZ3fN-UcFYmfJQ);

METAL, [http://www.sph.umich.edu/csg/abecasis/Metal](http://www.google.com/url?q=http%3A%2F%2Fwww.sph.umich.edu%2Fcsg%2Fabecasis%2FMetal&sa=D&sntz=1&usg=AFQjCNGATUxx5QilnP_YDyLTzhlJHTQ8FA) ;

SNAP, [http://www.broadinstitute.org/mpg/snap/](http://www.google.com/url?q=http%3A%2F%2Fwww.broadinstitute.org%2Fmpg%2Fsnap%2F&sa=D&sntz=1&usg=AFQjCNHfb9D61WaZjK9RgZOLXpVxDML2dA)

VEGAS, [http://gump.qimr.edu.au/VEGAS/](http://www.google.com/url?q=http%3A%2F%2Fgump.qimr.edu.au%2FVEGAS%2F&sa=D&sntz=1&usg=AFQjCNE8GwcuWo-WDL_9gZ1t2yONi0M-pQ);

LocusZoom, [http://csg.sph.umich.edu/locuszoom/](http://www.google.com/url?q=http%3A%2F%2Fcsg.sph.umich.edu%2Flocuszoom%2F&sa=D&sntz=1&usg=AFQjCNE9YpbQsVUVlH08LOReSfiuqMXiIw)

**Supplementary Information references**

1. (1989) The Atherosclerosis Risk in Communities (ARIC) Study: design and objectives. The ARIC investigators. Am J Epidemiol 129: 687-702.

2. Willett WC, Sampson L, Stampfer MJ, Rosner B, Bain C, et al. (1985) Reproducibility and validity of a semiquantitative food frequency questionnaire. Am J Epidemiol 122: 51-65.

3. Stevens J, Metcalf P, Dennis B, Tell G, Shimakawa T, et al. (1996) Reliability of a food frequency questionnaire by ethnicity, gender, age and education. Nutrition Research 16: 735-745.

4. Laurie CC, Doheny KF, Mirel DB, Pugh EW, Bierut LJ, et al. (2010) Quality control and quality assurance in genotypic data for genome-wide association studies. Genet Epidemiol 34: 591-602.

5. Price AL, Patterson NJ, Plenge RM, Weinblatt ME, Shadick NA, et al. (2006) Principal components analysis corrects for stratification in genome-wide association studies. Nat Genet 38: 904-909.

6. Yeager M, Chatterjee N, Ciampa J, Jacobs KB, Gonzalez-Bosquet J, et al. (2009) Identification of a new prostate cancer susceptibility locus on chromosome 8q24. Nat Genet 41: 1055-1057.

7. Colditz GA, Hankinson SE (2005) The Nurses' Health Study: lifestyle and health among women. Nat Rev Cancer 5: 388-396.

8. Willett WC (1998) Nutritional Epidemiology. New York: Oxford University Press.

9. Cornelis MC, El-Sohemy A (2007) Coffee, caffeine, and coronary heart disease. Curr Opin Lipidol 18: 13-19.

10. Korn JM, Kuruvilla FG, McCarroll SA, Wysoker A, Nemesh J, et al. (2008) Integrated genotype calling and association analysis of SNPs, common copy number polymorphisms and rare CNVs. Nat Genet 40: 1253-1260.

11. Qi L, Cornelis MC, Kraft P, Stanya KJ, Linda Kao WH, et al. (2010) Genetic variants at 2q24 are associated with susceptibility to type 2 diabetes. Hum Mol Genet 19: 2706-2715.

12. Hunter DJ, Kraft P, Jacobs KB, Cox DG, Yeager M, et al. (2007) A genome-wide association study identifies alleles in FGFR2 associated with risk of sporadic postmenopausal breast cancer. Nat Genet 39: 870-874.

13. Curhan GC, Taylor EN (2008) 24-h uric acid excretion and the risk of kidney stones. Kidney Int 73: 489-496.

14. Chu NF, Spiegelman D, Yu J, Rifai N, Hotamisligil GS, et al. (2001) Plasma leptin concentrations and four-year weight gain among US men. Int J Obes Relat Metab Disord 25: 346-353.

15. Ridker PM, Chasman DI, Zee RY, Parker A, Rose L, et al. (2008) Rationale, design, and methodology of the Women's Genome Health Study: a genome-wide association study of more than 25,000 initially healthy american women. Clin Chem 54: 249-255.
